# Development of high-performance nickel-based catalysts for production of hydrogen and carbon nanotubes from biogas

**DOI:** 10.1038/s41598-022-19638-y

**Published:** 2022-09-07

**Authors:** Supanida Saconsint, Nonthicha Sae-tang, Atthapon Srifa, Wanida Koo-Amornpattana, Suttichai Assabumrungrat, Choji Fukuhara, Sakhon Ratchahat

**Affiliations:** 1 Department of Chemical Engineering, Faculty of Engineering, Mahidol University, Nakhon Pathom, 73170 Thailand; 2 Center of Excellence in Catalysis and Catalytic Reaction Engineering, Department of Chemical Engineering, Faculty of Engineering, Chulalongkorn University, Bangkok, 10330 Thailand; 3 Department of Applied Chemistry and Biochemical Engineering, Graduate School of Engineering, Shizuoka University, Shizuoka, 432-8561 Japan

**Keywords:** Energy science and technology, Engineering, Materials science, Nanoscience and technology

## Abstract

Selecting a suitable catalyst for implementing the simultaneous production of hydrogen-rich syngas and multi-walled carbon nanotubes through the integration of dry reforming and methane decomposition reactions has recently gained great interests. In this study, a series of bimetallic (NiMo/MgO) and trimetallic (CoNiMo/MgO, FeNiMo/MgO, CoFeMo/MgO) catalysts was prepared and evaluated for a catalytic activity of CH_4_ and CO_2_ conversions of biogas in a fixed bed reactor at 800 °C and atmospheric pressure. Among the investigated catalysts, the bimetallic NiMo/MgO catalyst showed the outstanding catalytic performance with 86.4% CH_4_ conversion and 95.6% CO_2_ conversion as well as producing the highest syngas purity of 90.0% with H_2_/CO ratio = 1.1. Moreover, the characterization of the synthesized solid products proved that the well-aligned structured morphology, high purity, and excellent textural properties of CNTs were obtained by using NiMo/MgO catalyst. On the other hand, using trimetallic catalysts which have the composition of Co and Fe leads to the severe deactivation. This could be attributed the catalyst oxidation with CO_2_ in biogas, resulting in the transformation of metals into large metal oxides. The integrative process with NiMo/MgO catalyst is regarded as a promising pathway, which has a high potential for directly converting biogas into the high value-added products and providing a green approach for managing the enormous amounts of wastes.

## Introduction

Nowadays, the emissions of carbon dioxide (CO_2_), methane (CH_4_), and other greenhouse gases have all increased in the earth’s atmosphere more than at any time in the last 800,000 years. This is because of the dramatic growth of the world population and industrialization, resulting in huge energy consumption^1^. Fossil fuel combustion is found to be one of the greatest emitters of carbon dioxide which is a primary contribution of greenhouse gases that contributed to global warming, leading to unpredictable and negative changes in the environment^2^. These changes seem to become even crueler as time passes. Accordingly, to overcome these problems, renewable energy sources have drawn great interest in the research and development since they promise to reduce the environmental pollution while also increase the domestic energy supplies^3^.

Unlike other forms of renewable energy sources, biogas offers a very attractive route to utilize certain categories of biomass for meeting partial energy needs in both rural and industrial areas^4^. Biogas is a mixture primarily comprised of two components (e.g., CH_4_ and CO_2_) and is typically useful in a variety of ways including as vehicle fuel and to generate heat, electricity, combined heat, and power^5^. Another interesting option for utilizing biogas is to convert it directly into syngas (H_2_ and CO), which can be produced in different ways such as steam or partial oxidation reforming of methane (SRM or POM). Dry reforming of methane (DRM) as presented in Eq. (1), has been regarded as a favorable process to produce syngas from biogas because of its environmental and economic advantages^6,7^. It can provide a mixture of syngas with an H_2_/CO ratio close to unity which can be further beneficially upgraded to produce high-value-added products and is crucial for mitigating the greenhouse gas emissions^6^. Nevertheless, the coking issue is the main challenge for DRM because it can cause catalyst deactivation. Thus, a number of studies have been discussed on how to prevent carbon formation^8–10^. In this work, we proposed an approach to overcome this momentous concern by engineering the deposited carbon into carbon nanomaterials such as carbon nanotubes (CNTs) that can be also produced through catalytic decomposition of methane (CDM) as shown in Eq. (2). Since CNTs have raised a broad interest in the fields of science and technology. Because of their extraordinary physical and chemical properties, they can be used for a wide range of applications including energy storage^11^, electronic^12^, biomedical^13^, and environmental^7^.

1 $$\begin{array}{*{20}c} {{\text{CH}}_{{4}} {\text{ + CO}}_{{2}} { } \to {\text{ 2CO + 2H}}_{{2}} } & {\Delta {\text{H}}_{{{\text{298K}}}} { = + 247}\,{\text{kJ/mol}}} \\ \end{array}$$

2 $$\begin{array}{*{20}l} {{\text{CH}}_{{4 }} \to {\text{ C + H}}_{{2}} } \hfill & {\Delta {\text{H}}_{{\text{298K }}} { = + 74}{\text{.9 }}\,{\text{kJ/mol}}} \hfill \\ \end{array}$$

In comparison with the noble metals, such as Rh, Ru, Pt, Pd, and Ir, from a practical point of view, the supported transition metals, especially Ni catalysts have been extensively applied as a promising alternative for large-scale use because of their comparable catalytic activity and relatively low cost^14–16^. Figueredo et al*.*^17^ reported that Ni catalyst supported on perovskite-type LaAlO_3_ could reach the maximum values of CH_4_ and CO_2_ conversions of 94.0 and 85.1% at 700 °C, respectively. Moreover, methane conversion can be significantly improved with the increased Ni content and the reaction temperature, as reported in the literatures^17–19^. Co and Fe catalysts have also been studied^20–22^. It has been observed that the performance of Co supported catalysts exhibited very similar to that of Ni-based catalysts^23,24^. Meanwhile, Fe-based catalysts showed a lower activity than those catalysts containing Ni or Co, but they have received more attention than Co catalysts due to their economical and environmentally friendly advantages. Besides, they can provide the interesting characteristics and can be used at elevated temperatures^25,26^.

Even though they showed the superior performance, the activity of the catalyst has dropped rapidly due to the carbon-deposition-induced deactivation and subsequent sintering from the agglomeration of the particles at high reaction temperatures, which are still considered to be the main challenge for the DRM procedure^16^. Therefore, to increase the durability of the catalyst, most research studies have concentrated on the inclusion of a second metal to form bimetallic catalysts. Kutteri et al*.*^24^ adopted Ni, Co, and Fe to examine the performance of mono and bimetallic (Ni–Co, Ni–Fe, and Co–Fe) catalysts over SiO_2_ support for methane decomposition. The results revealed that all bimetallic catalysts have been found to be more active and stable than the single metal catalysts ones. This may be attributed to the formation of bimetallic alloys during the reaction as reported by Pudukudy et al*.*^27^. They have successfully used the set of novel Ni, Co, and Fe based bimetallic catalysts supported over SBA-15 to improve the activity and stability. Other metals, such as Mo, Sn, Cu, and Ce can be also used as a promoter in combination with active metals to further enhance the catalytic properties of the catalysts^23,28^. These authors^29,30^ studied the synergistic effect of the mixture between Co and Mo. They claimed that only Co alone did not catalyze the growth of SWCNTs. By contrast, alloying Mo in Co-based catalyst with an appropriate proportion can efficiently increase the yield and the selectivity towards the synthesis of high-quality CNTs. Moreover, it has been published that the addition of 5–10% Mo to Co/MgO catalyst raised the carbon yield about 20 times higher than usual and also extended the lifespan of the catalyst for the CNTs growth even after 2 h of synthesis time^31^. In similar findings, Awadallah et al*.*^31,32^ employed the Ni–Mo and Co–Mo/Al_2_O_3_ as catalysts with different metal loadings for the decomposition of natural gas into H_2_ and CNTs production. They found that all catalysts exhibited the excellent activity and durability up to 9 h without deactivation, suggesting that a small amount of Mo addition can keep the catalyst being active for a long time.

In the DRM process, another important component that affects the catalytic performance is catalyst supports, which are beneficial for anti-sintering ability, mechanical strength, and large specific surface area^16^. Many efforts have been devoted to investigate MgO and Al_2_O_3_ as support materials because of their availability, high thermal stability, and low cost^33–36^. Takenaka et al*.*^37^ determined the activity and lifetime of the Co catalysts supported on different supports (MgO, Al_2_O_3_, SiO_2_, and TiO_2_), and the results showed that the Co/Al_2_O_3_ and Co/MgO were more effective catalyst than those of Co/SiO_2_ and Co/TiO_2_ which could be explained by the crystallite size of Co metal particles in Co/Al_2_O_3_ and Co/MgO have diameters in the range of 10–30 nm that more easily to form CNFs, while the larger ones (> 30 nm) were inactive for methane decomposition reaction.

Therefore, in this study, we attempted to find a suitable catalyst for implementing the simultaneous production of hydrogen-rich syngas and multi-walled carbon nanotubes through DRM and CDM reactions by preparing the MgO-supported bi-, tri-metallic catalysts (NiMo/MgO, CoNiMo/MgO, FeNiMo/MgO, and CoFeMo/MgO) to combine their individual advantages, and investigated the effect of introducing a third metal on the performance of the catalysts and the properties of the synthesized CNTs.

## Experimental

### Catalyst preparation

The bimetallic NiMo and the trimetallic CoNiMo, FeNiMo, and CoFeMo/MgO catalysts were prepared by wetness impregnation method using the solution of metal precursors (Sigma Aldrich) such as Ni(NO_3_)_2_·6H_2_O, Co(NO_3_)_2_·6H_2_O, Fe(NO_3_)_3_·9H_2_O, and (NH_4_)_6_Mo_7_O_24_·4H_2_O. The metal loading was fixed at 30 wt.%. Typically, the metal precursors with a mass ratio of 1:1 for bimetallic catalyst and 1:1:1 for trimetallic catalyst was dissolved in DI water to get a completely mixed solution. This mixture was then added dropwise to MgO nano-powder (Merk) at room temperature while stirring to form a homogenous slurry, which was then evaporated on a hotplate at 80 °C till obtaining dried catalysts powder. The resulting catalysts powder was further calcined at 500 °C with a ramp rate of 10 °C/min in a muffle furnace for 3 h.

### Characterization

The calcined, reduced, and spent catalysts were characterized. The X-ray diffraction was conducted to examine the crystallinity and crystalline phases in calcined, reduced and spent catalysts using X-ray diffractometer (XRD, Bruker, D2 Phaser). The diffraction angle ranged from 2θ = 10° to 80°. The morphology and particle-sized distribution of synthesized CNTs were identified by a Field-emission scanning electron microscope (FE-SEM, JEOL, JSM-7610F) at the operating voltage of 10 kV. The internal structure of nano carbon deposited on the spent catalyst was characterized by a Field-emission transmission electron microscope (HR-TEM, JEOL, JEM-3100, Japan) at the operating voltage of 300 kV. The purity of the synthesized carbon nanotube was determined by thermogravimetric analysis (TGA, Metteler Toledo, TGA/DSC1) with an oxygen gas flow rate of 50 ml/min and a heating rate of 10 °C/min from room temperature to 800 °C. The hydrogen programmed reduction (H_2_-TPR, BEL JAPAN, BELCAT-B) method was used to assess the reducibility and metal-supported interaction of calcined catalysts. Prior to measurements, 100 mg of calcined catalysts was pretreated at 200 °C for 1 h under a He flowrate of 30 ml/min. Afterward, the H_2_-TPR profile was obtained using a mixture of 5% H_2_/Ar flowing at a rate of 30 ml/min with the sample heated from 100 to 900 °C with a fixed heating rate of 10 °C/min. The chemical composition of the reduced catalysts was analyzed by X-ray fluorescence (XRF, S8 TIGER, Series 2). The graphitic and disorder carbon of synthesized CNTs was examined by Raman spectrophotometer (PerkinElmerÒ Spectrum™ GX). The textural properties of spent catalysts were identified by the N_2_ sorption measurement (Micromeritics, TriStar II 3020).

### Production process

**Figure 1:**
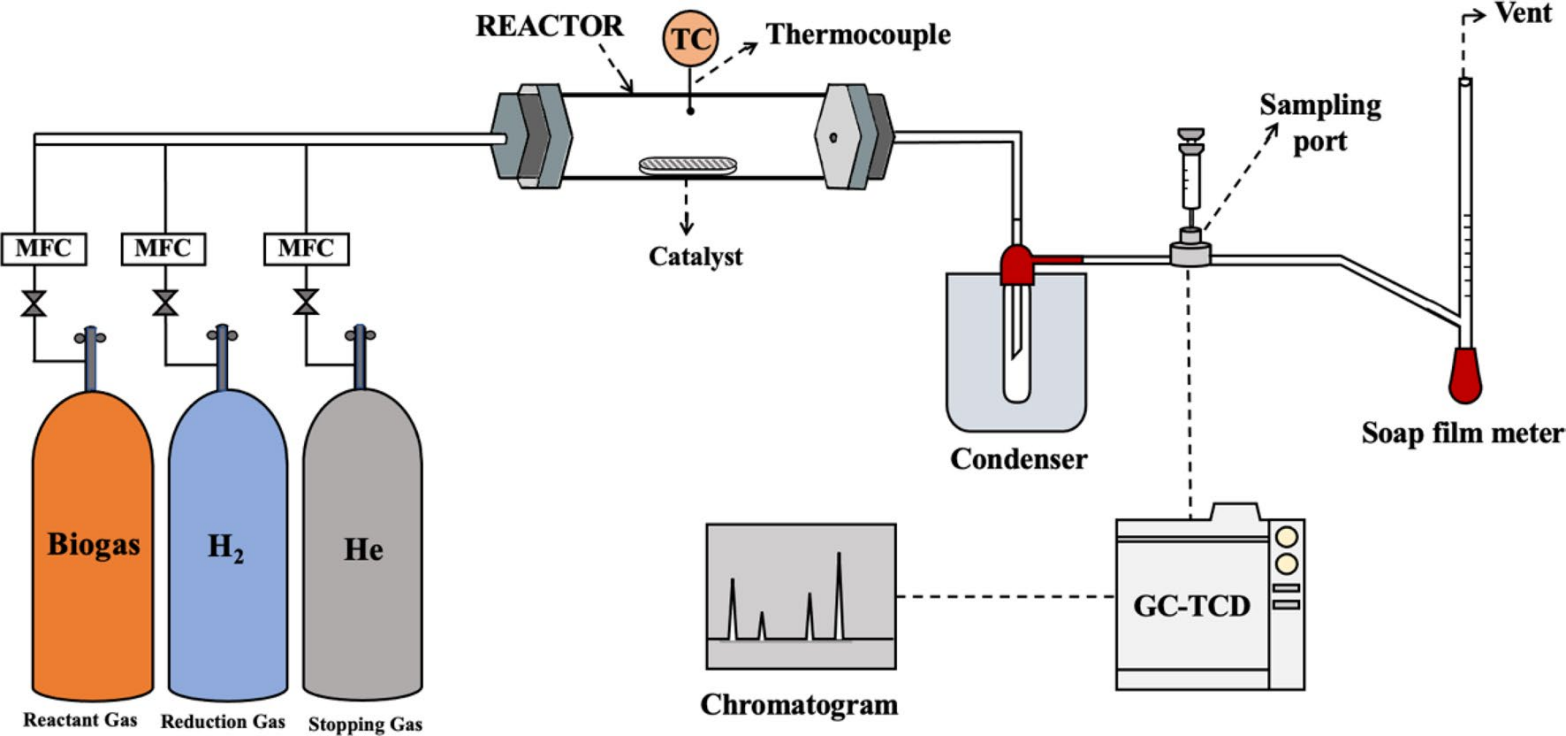
Schematic diagram of a process for directly converting biogas into hydrogen-rich syngas and carbon nanotubes.

The evaluation of the catalytic activity was performed under atmospheric pressure. The 0.5 g catalyst held on a quartz boat (15 mm width, 100 mm length, 8 mm height) was inserted at the center of a fixed-bed horizontal quartz reactor (26 mm I.D. and 1100 mm length) with PID temperature controller (KP1000 series, Chino) and Type-K thermocouple inserted at the center of the reactor. The experimental apparatus is schematically shown in Fig. 1. Prior to the reaction, the catalyst was reduced by H_2_ (75 ml/min) with a ramp rate of 10 °C/min until the temperature reached 1000 °C. Once the reactor temperature reached 1000 °C, biogas with a composition of 60% CH_4_ and 40% CO_2_ was continually fed into the reactor at the flow rate of 400 ml/min, corresponding to GHSV = 48,000 ml/g-h. During the reaction, the gas composition of the outlet stream was examined every 10 min using Gas chromatograph (TCD, Shimadzu, GC-2014). The volumetric flow rate of the inlet and outlet streams was controlled and calibrated by a mass flow controller (HORRIBA METRON, S48-32HMMT) and a soap bubble meter, respectively. After 3 h of the reaction time, the biogas was switched to the He flow at 50 ml/min to cool down the reactor to room temperature. The spent catalyst with deposited carbon was then weighed and collected in a desiccator for further characterization.

### Product analysis

The catalytic performance of the bimetallic and trimetallic catalysts in the integration of dry reforming.

3 $$X_{{{\text{CH}}_{{4}} }} (\% ) = \frac{{[CH_{4} ]_{in} \times F_{in } - [CH_{4} ]_{out} \times F_{in} }}{{[CH_{4} ]_{in} \times F_{in} }} \times 100$$

4 $$X_{{{\text{CO}}_{{2}} }} (\% ) = \frac{{[CO_{2} ]_{in} \times F_{in } - [CO_{2} ]_{out} \times F_{in} }}{{[CO_{2} ]_{in} \times F_{in} }} \times 100$$

For the gas analysis, the effluent gas of the outlet stream, H_2_ purity, and H_2_/CO ratio were calculated using Eqs. (5, 6), where *F*_*i*_ is the flow rate of component *i*, [*i*] represents the concentration of CH_4_, CO_2_, H_2_ or CO. *F*_in_ and *F*_out_ are the total gas volumetric flow rate of inlet and outlet from the reactor, in term of (ml/min). The production yield, purity of synthesized CNTs and gas hourly space velocity were calculated according to Eqs. (7–10).

5 $$F_{i} \left( {{\text{ml}}/{\text{min}}} \right) \, = \, \left[ i \right]_{{{\text{out}}}} \times F_{{{\text{out}}}}$$

6 $${\text{syngas}}\,{\text{ purity }}\left( \% \right) \, = \frac{{([H_{2} ]_{out} + [CO]_{out} ) \times F_{out} }}{{([CH_{4} ]_{out} + [CO_{2} ]_{out} + [H_{2} ]_{out} + [CO]_{out} ) \times F_{out} }} \times 100$$

7 $${\text{CNTs}}\,{\text{ gram}}\,{\text{ yield }}\left( {{\text{gCNTs}}/{\text{gCat - h}}} \right) \, = \frac{{m_{ product} - m _{catalyst} }}{{m _{catalyst} \times time}}$$

8 $${\text{Percent}}\,{\text{ yield}}\,{\text{ of}}\,{\text{ CNTs }}\left( \% \right) \, = \frac{{m_{ product} - m _{catalyst} }}{{m _{catalyst,in} }} \times 100$$

9 $${\text{CNTs}}\,{\text{ purity }}\left( \% \right) \, = \frac{{m_{ carbon} }}{{m _{product} }} \times 100$$

10 $${\text{GHSV }}\left( {{\text{ml}}/{\text{g}} - {\text{h}}} \right) \, = \frac{{F_{in} }}{{m _{catalyst} }}$$

whereas *F*_*in*_ is volumetric flow rate of reactant gas*, m*_*catalyst*_ is a mass of reduced catalyst, *m*_*product*_ is a mass of solid product including catalyst and CNTs, *m*_*carbon,in*_ is a mass of methane from inlet stream.

## Results and discussion

### Characterization of catalyst

All catalysts were prepared by wetness impregnation method. The total loading of metals (Ni, Co, Fe, and/or Mo) on MgO was fixed at 30 wt.%. The equal mass ratio of metals was used. Each catalyst was reduced by H_2_ (75 ml/min) before the characterization. The actual elemental composition of the reduced catalyst was analyzed by X-ray diffractometer (XRF) as shown in Table 1. The total metal loading of bimetallic catalyst was observed to be 31.6 wt.%. Likewise, the total metal loading of trimetallic catalyst is in the range of 30.0–30.8 wt.%. All catalyst results have a total metal loading that is quite close to the prescribed values. Furthermore, a trace of Ca impurity in all of the metal catalysts was detected with 0.2 wt.%.

**Table 1:** Elemental composition of reduced catalysts analyzed by XRF.

Catalysts	Ni (wt.%)	Co (wt.%)	Fe (wt.%)	Mo (wt.%)	MgO (wt.%)	Ca (wt.%)
NiMo	16.3	–	–	15.3	68.1	0.2
CoNiMo	10.6	10.2	–	10.0	69.0	0.2
FeNiMo	10.2	–	10.1	10.1	69.4	0.2
CoFeMo	–	10.1	10.0	9.9	69.9	0.2

**Figure 2:**
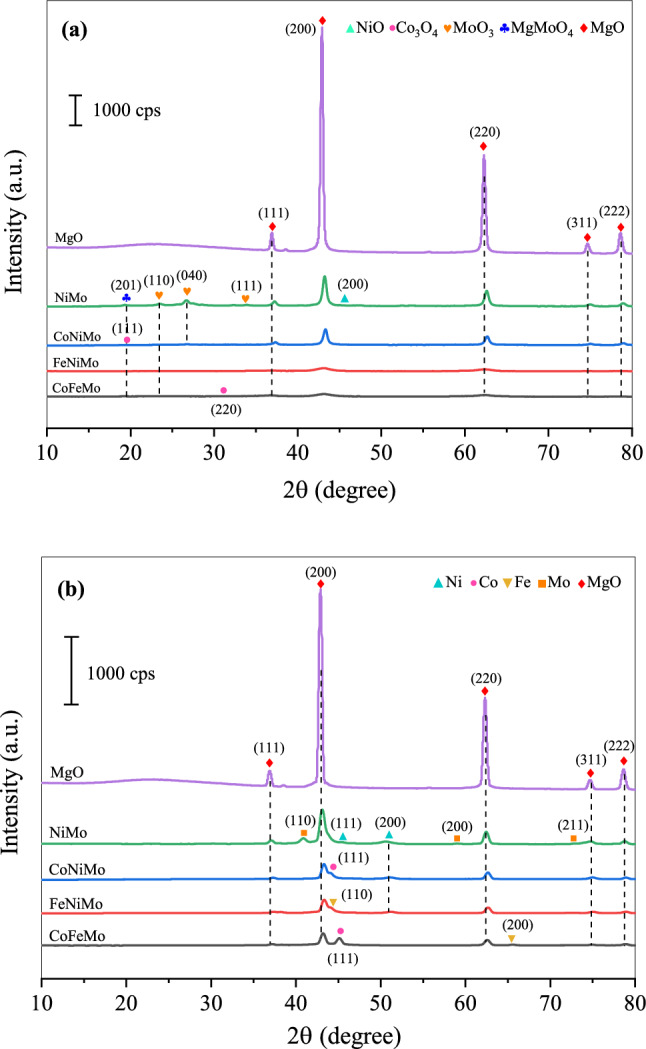
XRD patterns of the fresh catalysts after (**a**) calcination and (**b**) reduction processes.

X-ray diffraction (XRD) measurement was principally carried out to identify the crystalline phases arising during the process. The results of the contemporary catalysts analyzed by XRD after calcination at 500 °C for 3 h are depicted in Fig. 2a. For all samples, many diffraction peaks that mainly ascribed to magnesium oxide support (JCPDS 45-0946) were observed at 2θ = 36.96°, 42.92°, 62.29°, 74.59°, and 78.58°, which is perfectly in accordance with (111), (200), (220), (311), and (222) planes. However, the crystal phase of NiO (JCPDS 47-1049) was identified only in the pattern of NiMo/MgO catalyst, showing a higher number of particles consisting of Ni species on the surface of the support. Moreover, the Co_3_O_4_ phases (JCPDS 42-1467) are almost absent as identified in the XRD results profile of CoNiMo and CoFeMo/MgO catalysts. The possible reason is that it can be well dispersed in the support and/or too small to be detected. The appearance of MoO_3_ species (JCPDS 05-0508) in these catalysts are known to form due to the oxidation of Mo at temperatures above 500 °C^37^. That can be attributed to a high composition of molybdenum oxide phases on the catalysts surface. The reflection peaks of MgMoO_4_ (JCPDS 72-2153) are also detected with very low or less intensity in the diffraction pattern of all catalysts, which formed via interaction between MgO and MoO_3_ at a temperature higher than 400 °C^38^.

Figure 2b illustrates XRD diffractograms of fresh catalysts obtained after carrying out the activation process by reducing under certain conditions, along with the structural parameters analyzed by the XRD technique are presented in Table 2. It was noticed that the characteristic peak of MgO phases appears extensively in all catalysts. Also, it can be clearly seen that the MgO crystal domain size significantly diminished in all catalysts compared to the reduced MgO. In addition, the reflections attributed to metallic forms such as Ni (JCPDS 04-0850), Co (ICPDS 15-0806), Fe (JCPDS 65-4899), and Mo (ICSD 64-3962) were detected. In comparison, the crystallinity of metal Mo peaks for using bi-metallic NiMo/MgO catalyst presents a higher than other tri-metallic catalysts, revealing a high percentage of Mo in the catalyst. Meanwhile, no other diffraction patterns reflexed to their oxides were observed, expecting that metallic phases can be completely reduced into the activated catalyst by the usage of hydrogen reducibility at a high temperature.

**Table 2:** The crystal size of reduced MgO and reduced catalysts by XRD analysis.

Catalysts	Crystallite size (nm)				
	Ni (111)	Co (111)	Fe (110)	Mo (110)	MgO (200)
MgO	–	–	–	–	24.10
NiMo	7.46	–	–	12.28	18.09
CoNiMo	nd	8.67	–	nd	19.11
FeNiMo	nd	–	9.68	nd	16.60
CoFeMo	–	18.82	nd	nd	17.56

**Figure 3:**
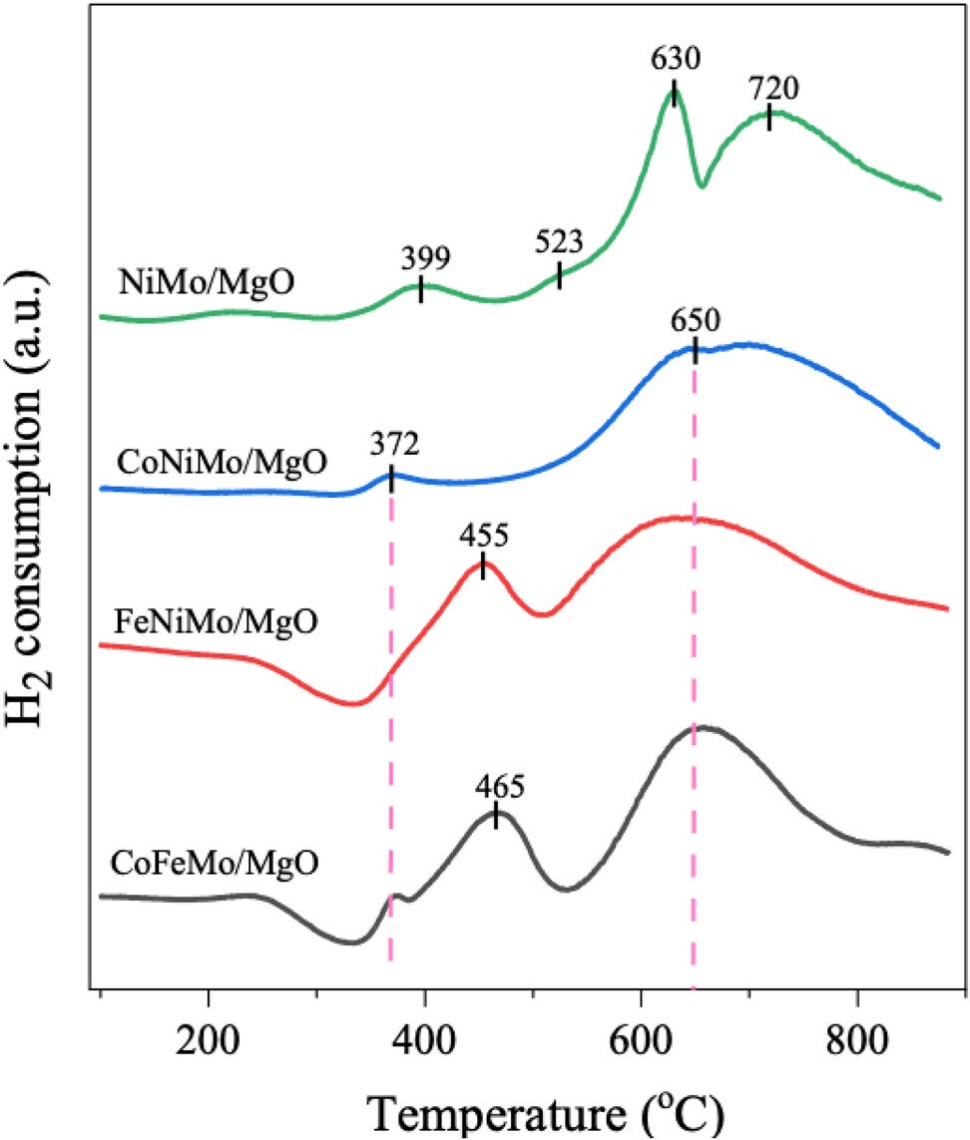
H_2_-TPR profiles of fresh calcined catalysts.

The reducibility of all the fresh calcined catalysts used in this study was determined by the H_2_-TPR technique and the results are shown in Fig. 3. For the bimetallic NiMo/MgO catalyst, the TPR profile illustrates two hydrogen consumption regions: in the first low-temperature region, between 300 and 600 °C, a broad peak with a slightly low intensity observed at 399 °C is assigned to the reduction of loosely attached NiO bound on the support surface, and the second, shoulder peak centered at 523 °C could be attributed to the reduction of NiO species strongly interacted with the support^37,39^. At the higher temperature region between ranges of 600–1000 °C, the profile shows the presence of two distinct peaks that appear centered at 630 and 720 °C, indicating two differentiated reduction processes. According to previous studies^40–42^, the former represents the reduction of octahedral Mo-oxo species (Mo^6+^ → Mo^4+^) together with the first reduction step of MgMoO_4_, and the following peak consumed in the high reduction temperature is associated with the second reduction step of MgMoO_4_ as well as the further reduction from tetrahedral Mo^6+^ to metallic Mo^0^ passing through the Mo^4+^ phase which has been reported that is more difficult to reduce than that of octahedral ones.

On the other hand, the H_2_ consumption peaks of the trimetallic with Co-containing catalysts appeared centered at temperatures of 372 °C are related to the reduction from Co_3_O_4_ to metallic Co^0^ phase. It is reported that if the size of these peaks is small, the relative amount of these crystals is very low. Thus, it can be said that the MgO surface held a small proportion of Co_3_O_4_^43,44^. Also, the peaks at a temperature range of 400–500 °C were detected in the catalysts composed of Fe content, which reflected the formation of metallic iron from the two-step reduction of magnetite as a sequence of Fe_3_O_4_ to FeO and Fe^21,41,45^. In addition, the TPR profiles of all trimetallic catalysts display a single broad peak with a maximum at around 650 °C which is deconvoluted into mainly two peaks corresponding to the reduction of MgMoO_4_ and Mo species as mentioned before. These peaks begin with temperatures higher than 500 °C and seem to continue even up to 900 °C, especially in the profiles of FeNiMo/MgO and CoFeMo/MgO catalysts. The broad tail at high temperature is probably due to the result of a larger amount of Fe oxides were not completely reduced to Fe^0^, indicating a strong interaction between iron species and the support^46^. This suggests that both catalysts needed temperatures higher than 900 °C for the full reduction into the metallic form.

### Evaluation of catalytic performance and durability

**Figure 4:**
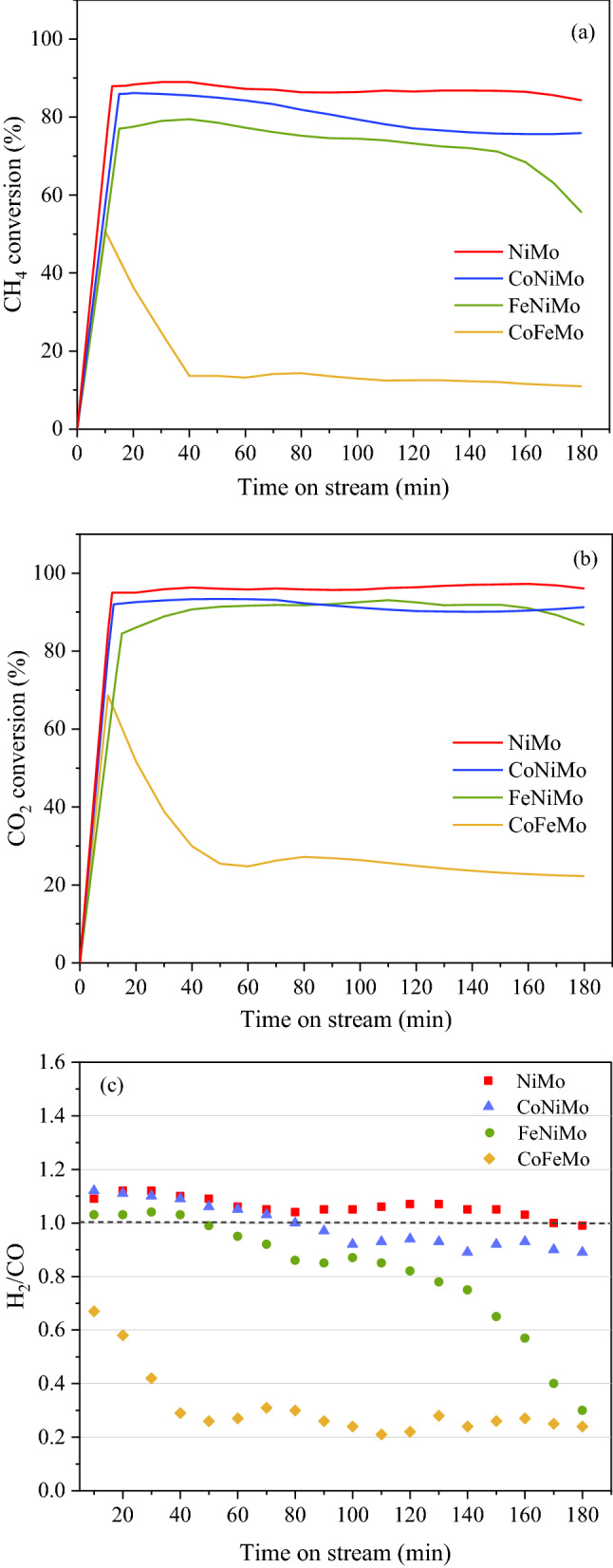
**(a)** CH_4_ conversion, **(b)** CO_2_ conversion, and **(c)** H_2_/CO ratio as a function of time on stream at 800 °C for 3 h, GHSV = 48,000 ml/gCat-h, over a bimetallic catalyst (NiMo) and trimetallic catalysts (CoNiMo, FeNiMo, and CoFeMo) supported on MgO.

The performance of bimetallic and trimetallic catalysts was evaluated under the integration of dry reforming catalytic decomposition of methane. All catalytic has been carried out in a fixed bed reactor at 800 °C, 1 atm under biogas (CH_4_:CO_2_ = 1.5:1) feeding, with a corresponding space velocity of GHSV = 48,000 ml/gCat-h. The feed gas conversion and the amount of H_2_ and CO product were calculated based on the measurement of the effluent gas composition of the outlet stream during the reaction for 3 h. In this process, the decomposition of CH_4_ on the catalyst surface leads to the production of H_2_ and carbon nanotubes (CNTs)^47,48^. Meanwhile, CO_2_ interacts with CH_4_ through a dry reforming reaction, which is accompanied by a parallel reaction that produces syngas (H_2_ + CO)^49^. As a result, the outlet stream effluent gas contains H_2_, CO, and a small amount of unconverted CO_2_ and CH_4_. Figure 4a,b shows the CH_4_ and CO_2_ conversion as a function of time on stream. It is clearly observed that the coexistence of Ni and Mo reveals exceptional performance with achieving higher CH_4_ conversion of 86.4% and CO_2_ conversion of 95.6% in NiMo catalyst as well as showing excellent stability over 3 h. Besides, H_2_-rich syngas was produced over NiMo/MgO catalyst having H_2_/CO ratio of 1.1. In addition, the syngas purity of 90.0% was obtained as listed in Table 3. Meanwhile, the addition of Co and Fe in the catalyst is found to be that the conversion of CH_4_ and CO_2_ decreases gradually. This occurrence may imply that the catalyst surface of Co and Fe would have undergone oxidation while exposure to CO_2_ in biogas led to the catalyst deactivation^26,50–52^.

**Table 3:** Summary of CNTs yield, CNTs purity, H_2_/CO ratio, and Syngas purity with different catalysts.

Catalyst	Yield (gProduct/gCat-h)	CNTs yield (%)^a^	Purity (%)^b^	H_2_/CO ratio (−)	Syngas purity (%)
NiMo	2.60	11.8	84.0	1.1	90.0
CoNiMo	2.27	9.9	83.9	1.0	84.1
FeNiMo	1.13	4.1	75.6	0.8	79.4
CoFeMo	0.28	n.d	n.d	0.3	21.8

^a^Calculation based on methane in feedstock.

^b^Analysis by TGA.

Table 4 compares the performance of NiMo/MgO catalyst, with respect to Ni-based catalysts from the previous reports for converting biogas in various circumstances. In the case of the CH_4_/CO_2_ ratio equal to 1.5/1, NiMo/MgO catalyst in the present study outperforms the competition in CH_4_ and CO_2_ conversions. Our catalyst possesses outstanding performance for syngas production having the H_2_/CO ratio > 1, a resource for producing high-value products such as methanol and liquid hydrocarbons by Fischer–Tropsch synthesis^53,54^. Furthermore, NiMo/MgO catalyst can perform superior for the formation of valuable carbon nanomaterials without encountering the deactivation of the catalyst during the tested time. These findings assure that our catalyst is regarded as a promising catalyst for biogas transformation into syngas and carbon nanomaterials through the integrative process of DRM and CDM. However, as indicated in Table 4, the concentrations of CH_4_ and CO_2_ in biogas are another important factor in the integration process of DRM and CDM, which influences the biogas conversion and the syngas production.

**Table 4:** Comparison of the catalytic performance of Ni-based catalysts in producing syngas from biogas.

Catalyst	CH_4_/CO_2_	GHSV (ml/g-h)	Temperature (°C)	CH_4_ conversion (%)	CO_2_ conversion (%)	H_2_/CO ( −)	Ref
10 wt.%Ni–3 wt.%Pt/Al_2_O_3_	1/1	600	800	90.0	95.0	0.90	^55^
7 wt.%Ni–3 wt.%Co/LaAl	1/1	6000	800	93.7	94.0	0.97	^56^
Ni-Rh/Ce-Al_2_O_3_	1.5/1	60,000	800	60.1	94.4	1.0	^57^
Ni/Ce-La-Al_2_O_3_	1.5/1	120,000	800	69.0	94.5	0.9	^58^
Ni–Sn/Ce-Al_2_O_3_	1.5/1	30,000	800	63.1	97.5	1.0	^59^
NiMo/MgO	1.5/1	48,000	800	86.4	95.6	1.1	This work

### Characterization of CNTs products

CNTs generated from the decomposition of CH_4_ were examined for yield, purity, crystallinity, graphitization, morphology, and textural properties. Table 3 presents the production yield of CNTs in terms of gProduct/gCat-h was synthesized over bimetallic (NiMo/MgO) and trimetallic (CoNiMo/MgO, FeNiMo/MgO, CoFeMo/MgO). The bimetallic NiMo/MgO catalyst exhibited high performance for the CNTs production of 2.60 gProduct/gCat-h than all trimetallic catalysts. The production yield of CNTs obviously that the cohabitation of Ni and Mo particles drastically encouraged the creation of CNTs through CDM, Eq. (2)^60^ as compared to that obtained without Ni catalyst^60^ as compared to that obtained without Ni catalyst. On the contrary, the addition of Co and Fe to the catalyst induced a significant abatement yield of synthesized carbon nanotubes. This could be owing to the metals Co and Fe in biogas being easily oxidized by CO_2_ and forming metal oxides^26,50–52^.

**Figure 5:**
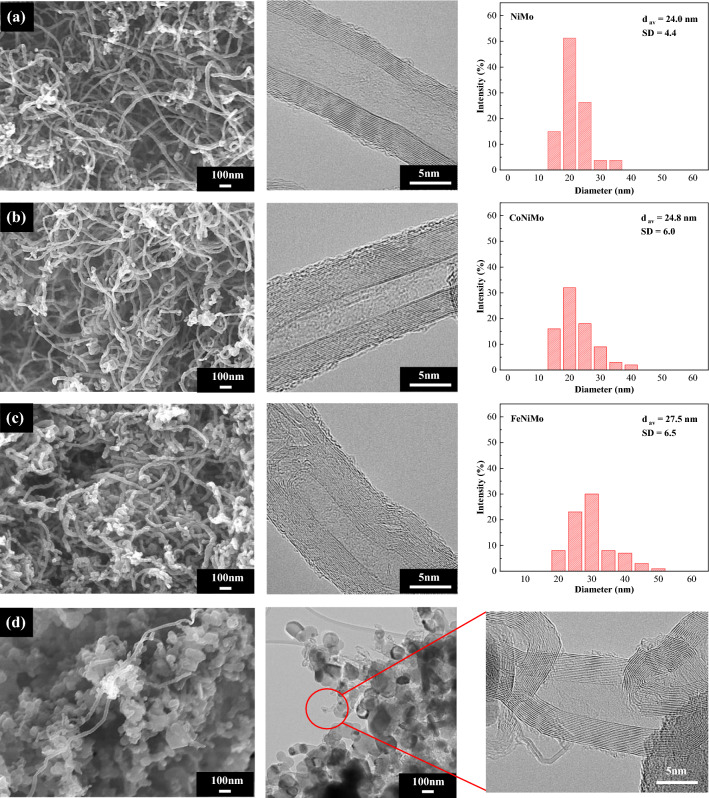
FE-SEM (× 50 k, 10 kV), HR-TEM (× 600 k, 300 kV) micrographs, and particle size distribution of synthesized CNTs over (**a**) NiMo, (**b**) CoNiMo, (**c**) FeNiMo, and (**d**) CoFeMo catalysts supported on MgO (Reaction conditions: 800 °C, 1 atm, 3 h.).

Figure 5 provides more insight into the morphological differences of the deposited carbon over various catalysts used, as well as the analyzing parameters of synthesized CNTs which were calculated from FE-SEM and HR-TEM images are presented in Table 5. From the SEM images, it was clearly observed that the presence of condensed nanocarbon was observed on the catalyst surface, especially on catalysts that contain Ni metal particles while a small amount of CNTs detected on the surface of the catalyst with the absence of Ni (Fig. 5d). This indicates the high activity and capability of producing CNTs by using Ni-based catalysts. In addition, TEM images as shown in Fig. 5, confirm the formation of multi-walled carbon nanotubes (MWCNTs) on all catalysts used in this study. Also, it is obvious that the addition of Fe in the catalyst forming the bamboo type in carbon nanostructures^21,25^ which may be useful in some practical applications, such as hydrogen storage, electrochemical capacitors, and lithium-ion batteries^61^.

**Table 5:** Summary of morphology analysis of CNTs synthesized from different catalysts.

Parameter	Catalyst				CNTs (Bayer®)
	NiMo	CoNiMo	FeNiMo	CoFeMo	
Type of CNTs	MWCNTs	MWCNTs	MWCNTs	MWCNTs	MWCNTs
Distribution (nm)	16–37	15–43	16–47	31–32	10–30
d_av_ (nm)^a^	24.0	24.8	27.5	31.4	22.0
sd.^b^	4.4	6.0	6.5	n.d	3.3
No. of wall	13–25	15–25	16–28	n.d	9–24

^a^Diameter of CNTs calculated by mode.

^b^Standard deviation of CNTs diameter.

From Fig. 5 and Table 5, it was found that the narrowest and smallest carbon nanotubes can be achieved over NiMo catalyst without any further combination with other transition metals. Meanwhile, the addition of Fe and Co apparently gives the higher range of the wall numbers and diameters of CNTs. This might be attributed to the agglomeration of oxide species (Fe_3_O_4_ and Co_3_O_4_) on the catalyst surface which leads to an increase in metal particle size, consequently, large CNTs are produced^44,62^.

**Figure 6:**
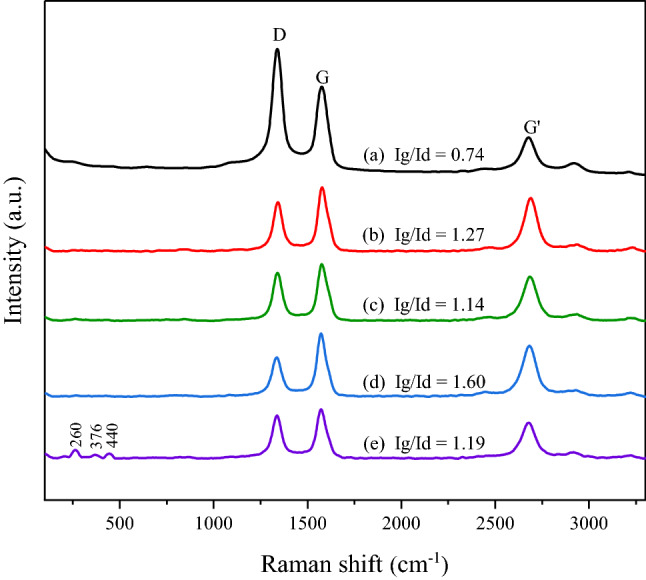
Raman spectra of the CNTs synthesized over different catalysts at 800 °C for 3 h: (**a**) CNTs_commercial (Bayer^®^), (**b**) CNTs_NiMo, (**c**) CNTs_CoNiMo, (**d**) CNTs_FeNiMo, and **(e)** CNTs_CoFeMo.

The graphitization of all synthesized CNTs produced onto bimetallic and trimetallic catalysts at 800 °C was evaluated by Raman spectroscopy. All of these spectra show three distinct peaks, including D-band, G-band, and G′-band. The D-band at around 1300–1400 cm^−1^ is associated with the structural defect and impurity which represents the disordered carbon or amorphous carbon deposited on the outer surface of carbon nanotubes^63^. The G-band at around 1500–1600 cm^-1^ is related to the tangential stretching mode of all pairs of *sp*^*2*^ atoms in both rings and chains which represents the graphitic carbon structure^61^, while G’-band observed at around 2600–2700 cm^−1^ is associated with the process of two-photon elastic scattering^64^. The appearance of both G-band and G’-band can be used as an indicator for assuring the formation of graphite nanotube. This data is in accordance with the morphology observation in Fig. 5. The relation between D-band and G-band in the aspects of intensity ratio Ig/Id is used to evaluate the quality of MWCNTs on the used catalyst, which the low value of the Ig/Id ratio (> 1) indicates a good degree of graphitization. The results from Raman analyze show that all synthesized CNTs (Ig/Id = 1.14–1.60) presented the Ig/Id ratio higher than commercial CNTs (Ig/Id = 0.74). As seen in Fig. 6, the synthesized CNTs over the FeNiMo catalyst had the highest graphitization carbon structure (Ig/Id = 1.60) due to Fe metal having a high carbon solubility^47^. Therefore, the addition of Fe into NiMo/MgO catalysts favors the formation of CNTs with a high graphitization and a small amount of defect on the wall surface, which corresponds to the TEM analysis shown in Fig. 5. On considering CoFeMo/MgO catalyst, the Raman shift appears at 260, 376, and 440 cm^−1^ reflecting the presence of MoO_3_. This peak shoulder could be confirmed the oxidation of Mo with CO_2_ on the surface catalyst, which may be converting Mo into MoO_3_^26^.

**Figure 7:**
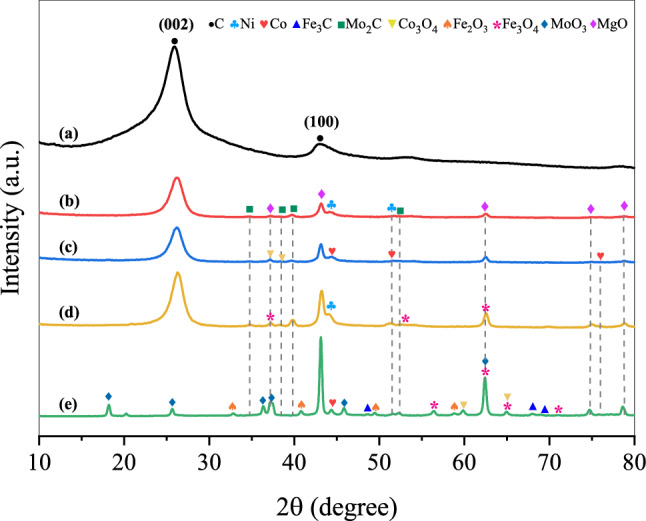
XRD patterns of (**a**) commercial CNTs (Bayer^®^) and synthesized CNTs over different catalysts at 800 °C for 3 h : (**b**) NiMo/MgO, (**c**) CoNiMo/MgO, (**d**) FeNiMo/MgO, and (**e**) CoFeMo/MgO.

Figure 7 displays XRD diffraction data for the CNTs generated after conducting in the isothermal test at 800 °C for 3 h. All the pattern results, besides the CoFeMo/MgO catalyst, the intensity of the 002 plane of the graphitic peaks at 2$\uptheta$ = 26.1° are more prominent compared to the relevant peaks that are assigned to metallic and other phases. This peak is known to quantify the crystallinity of carbon materials. However, in our study, there seems to be more associated with the formation of CNTs, where the sharp diffraction peak shows the high activity of the catalysts for achieving the carbon products, which confirms the higher yield of the carbon nanofilaments grown over these catalysts. The interlayer distance (*d*_*002*_) or d-spacing value derived from the XRD data can be used to the structural degree of synthesized CNTs as well, which was calculated by applying the Bragg’s equation ($d=nl/2\mathrm{sin}\theta$)^65,66^ and the results are presented in Table 6. It was found that the used catalysts have produced the CNTs with the interplanar spacing value in the range between 0.344 and 0.348 nm, which is very nearly the value obtained from commercial CNTs (0.343 nm). As shown in the table, the crystallite size of Ni, Fe, and Co particles decreased in all samples compared to the reduced catalysts except for the CoFeMo/MgO catalyst (Figs. 3b, 7). This could be implied that the portion of Ni in the catalyst makes the metal particles to be small, resulting in the increase of the active surface area thus improving the dispersion and catalytic activity of active nanoparticles.

**Table 6:** Structural parameters analyzed by XRD for carbon products (interplanar spacing (d_002_)), and other components (crystal size).

Samples	d (002)	Ni (111)	Co (111)	Fe (110)	Fe_3_C (202)	Mo_2_C (101)	Co_3_O_4_ (422)	Fe_3_O_4_ (511)
CNTs_NiMo	0.348	6.83	–	–	–	8.26	–	–
CNTs_CoNiMo	0.346	n.d	6.49	–	–	11.74	10.39	–
CNTs_FeNiMo	0.344	n.d	–	n.d	n.d	14.86	–	15.90
CNTs_CoFeMo	n.d	–	19.50	n.d	38.84	42.64	25.44	23.10

After the reaction, The Mo_2_C peaks slightly appeared for all cases, which have been known to be generated during the formation of CNTs via the carbide cycle mechanism^28^. In addition, the presence of MoO_3_ in the consumed CoFeMo/MgO catalyst can be ascribed to the oxidation of Co as mentioned above. It might be corresponded to the conversion of Mo into their oxides^26^. The several reflection peaks of Co_3_O_4_ and Fe_3_O_4_ are detected in the XRD pattern of the Co- and Fe-containing catalysts. This certifies that Co and Fe metallic phases could be oxidized with CO_2_ to form metal oxides. Besides, some spot of Fe_2_O_3_, and Fe_3_C peaks were also identified with very low intensities especially on the surface of the CoFeMo/MgO catalyst. This suggested that some Fe particles were possibly converted into Fe_2_O_3_, and Fe_3_C through the oxidation of Fe with carbon dioxide (CO_2_ + Fe → Fe_2_O_3_ + Fe_3_C). In order to better understand what exactly happens, another two approaches are performed, and latter being thoroughly discussed in “The influence of CO_2_ oxidation” section. Moreover, the bigger diameter of CNTs generated with the CoNiMo/MgO, FeNiMo/MgO, and CoFeMo/MgO catalysts can be regarded as the huge proportion of their metal oxide forms hold on the surface of catalysts, which are responsible for the growing of large diameter CNTs. Since the crystal size of oxide species is normally higher than its usual metallic form as obviously seen in Table 6 and TEM images in Fig. 7.

**Figure 8:**
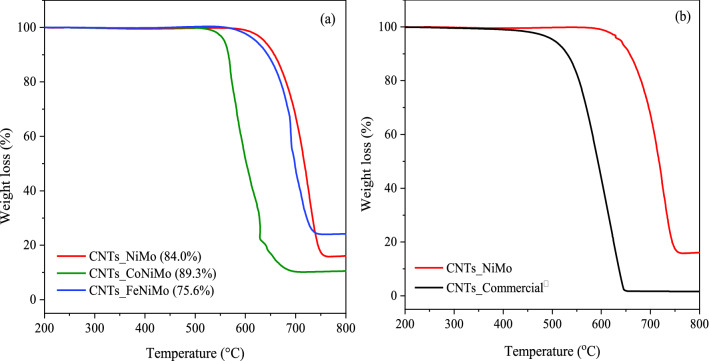
TGA curves of CNTs synthesized over spent catalysts (**a**) in this study (**b**) compared to commercial CNTs (Bayer^®^).

Thermal gravimetric analysis (TGA) was used to determine the quality and the purity of CNTs formed over these catalysts, and the results are depicted in Fig. 8. The weight loss is ascribed to the burning of deposited carbon in oxygen and then corresponds to the yield of solid carbon in the catalysts^67^. Thus, as can be seen in Fig. 8a, the largest weight loss of about 84% was obtained by using the NiMo/MgO catalyst, indicating the presence of high carbon content in the sample, and hence high purity CNTs was observed. The impurities in the CNTs sample were possibly attributed to the residual catalysts that remained after the reaction and other species including oxygen from CO_2_ which would possibly be contained in the samples. In general, the classic chemical technique for separating CNTs from other entities such as residual catalysts has been done by treating the CNTs sample with acid leaching^68^. Moreover, the purity of CNTs can be further increased by extending the reaction time as well, as reported in our previous work^26^. The TGA profiles of the carbonaceous materials deposited on all catalysts show similar oxidation behaviors with single-step degradation, regarding the absence of amorphous carbon which is closely related with their textural and structural properties. Additionally, a thorough analysis of all the results derived from TGA data illustrated a high inflection at temperatures up to 550 °C, reflecting that thermal stability and structural degree of CNTs had improved^67^. Particularly again in the case of the NiMo catalyst, the CNTs was produced with remarkably outstanding thermal stability better than the commercial as demonstrated in Fig. 8b. However, the purity of synthesized CNTs has lower than the commercial CNTs ones.

**Figure 9:**
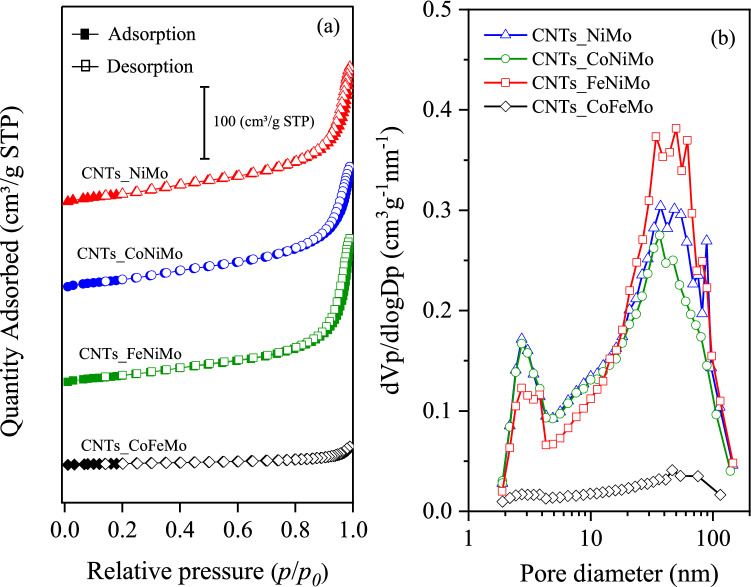
(**a**) N_2_ adsorption–desorption isotherms and (**b**) pore size distribution of synthesized CNTs over different catalysts with 800 °C.

The textural properties of CNTs were assessed by N_2_ sorption measurements, the data are listed in Table 7 and shown in Fig. 9 The highest surface area was obtained by synthesized CNTs over NiMo/MgO catalysts. In contrast, the addition of Co into NiMo/MgO catalysts is reduced the BET surface area of synthesized CNTs owing to the oxidation of Co leading to the deactivation of the catalyst. Additionally, the effect of the addition of Fe into NiMo/MgO catalysts results in a decrease in the BET surface area of synthesized CNTs due to Fe could be readily oxidized in exposure CO_2_ in Biogas, which could have caused the catalyst deactivation^50–52^. For these reasons, the synthesized CNTs over CoFeMo/MgO exhibit the lowest surface area. According to Fig. 9a, N_2_ absorption–desorption isotherms of all synthesized CNTs, present type IV isotherms according to IUPAC classification, indicating that the majority of porosity in the CNTs is mesoporous^69^. These results consistent with the mean pore size of synthesized CNTs is in 10–13 nm which is in the range of mesopore material (2–50 nm), as shown in Table 7. The hysteresis loops formed by the capillary condensation effect can be classified as H3 hysteretic loops, usually found in solids consisting of aggregates or agglomerates of particles forming slit-shaped pores, with a non-uniform size and/or shape^70^. The pore diameter distributions based on the BJH method as shown in Fig. 9b present the curves, which can be ascribed that all the synthesized CNTs exhibit a bimodal feature that has a pore size with a board distribution ranging from 2 to 150 nm, including a small pore-size fraction (2–5 nm) and a large pore-size fraction (5–150 nm).

**Table 7:** Summary of surface area and porosity of CNTs synthesized with different catalysts.

Sample	Isotherms	S_BET_ (m^2^/g)	V_meso_ (cm^3^/g)	V_total_ (cm^3^/g)	D_avg_ (nm)
CNTs_NiMo	Type IV	127.4	0.3223	0.3273	11.0
CNTs_CoNiMo	Type IV	124.8	0.2913	0.2957	10.2
CNTs_FeNiMo	Type IV	105.7	0.3382	0.3410	13.6
CNTs_CoFeMo	Type IV	18.4	0.0435	0.0445	11.2
CNTs_Bayer^®^	Type IV	199.8	0.2059	2.4442	48.9

### The influence of CO_2_ oxidation

**Figure 10:**
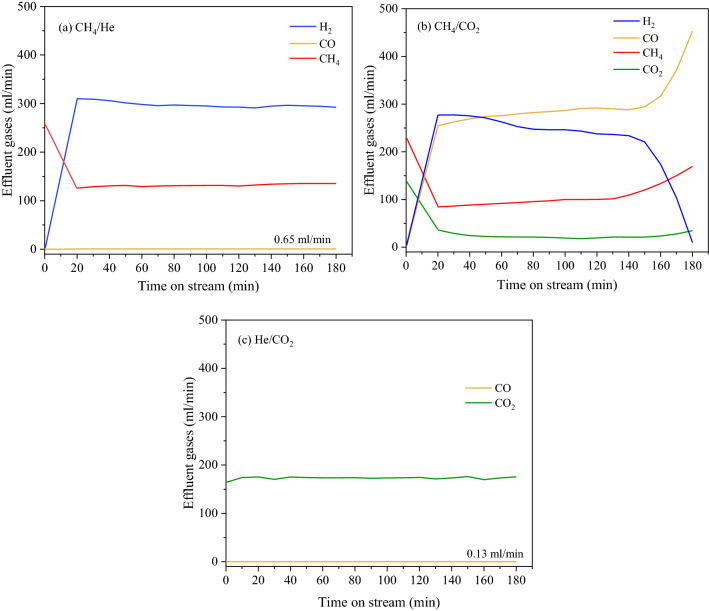
The composition of effluent gas at 800 °C for 3 h over FeNiMo/MgO catalyst under (**a**) CH_4_/He (**b**) CH_4_/CO_2_ (**c**) He/CO_2_, GHSV = 48,000 ml/gCat-h.

One aspect that the influence of CO_2_ oxidation was revealed in the former discussion. Herein, an effort is exerted to comprehend how CO_2_ oxidation influences Fe-based metal catalysts through performing under two different conditions including CH_4_/He (3/2), CH_4_/CO_2_ (3/2), and He/CO_2_ (3/2) feeding into the reactor. All this condition was operated over FeNiMo/MgO catalyst at 800 °C with the flow rate of 400 ml/min, corresponding to GHSV = 48,000 ml/gCat-h. The reaction time was set to 3 h. Figure 10 shows the effluent gas of the outlet stream for each condition. Considering the CH_4_ decomposition reaction as seen in Fig. 10a, H_2_ and CNTs are generally being product according to Eq. (2). Meanwhile, it is noteworthy that the CO concentration is slightly present in the effluent gas of the outlet stream. This observation can be clarified that the existence of iron oxide (hematite, Fe_2_O_3_) due to unaccomplished reduction under H_2_ at 1000 °C with a heating rate of 10 °C/min^71^ is reduced with CH_4_ to form CO according to Eq. (11)^72^. In this regard, CH_4_ is probably more effective than H_2_ as a reductant. For the condition under He/CO_2_, Fe could be readily oxidized with CO_2_ to form Fe_2_O_3_ and Fe_3_C which could have caused the activity loss according to Eq. (12). Furthermore, the CO composition was found to be slightly concentrated, as illustrated in Fig. 10c. This occurrence regarded as another form of Fe is being oxidized with CO_2_ to produce CO and Fe_3_O_4_ resulting in loss activity according to Eq. (13).

11 $${\text{Fe}}_{{2}} {\text{O}}_{{3}} + {\text{ 3CH}}_{{4}} ~ \to {\text{3CO }} + {\text{ 2Fe }} + {\text{ 6H}}_{{2}}$$

12 $${\text{3CO}}_{{2}} + {\text{ 13Fe}} ~ \to {\text{2Fe}}_{{2}} {\text{O}}_{{3}} + {\text{ 3Fe}}_{{3}} {\text{C}}$$

13 $${\text{4CO}}_{{2}} + {\text{ 3Fe}} ~ \to {\text{Fe}}_{{3}} {\text{O}}_{{4}} + {\text{ 4CO}}$$

**Figure 11:**
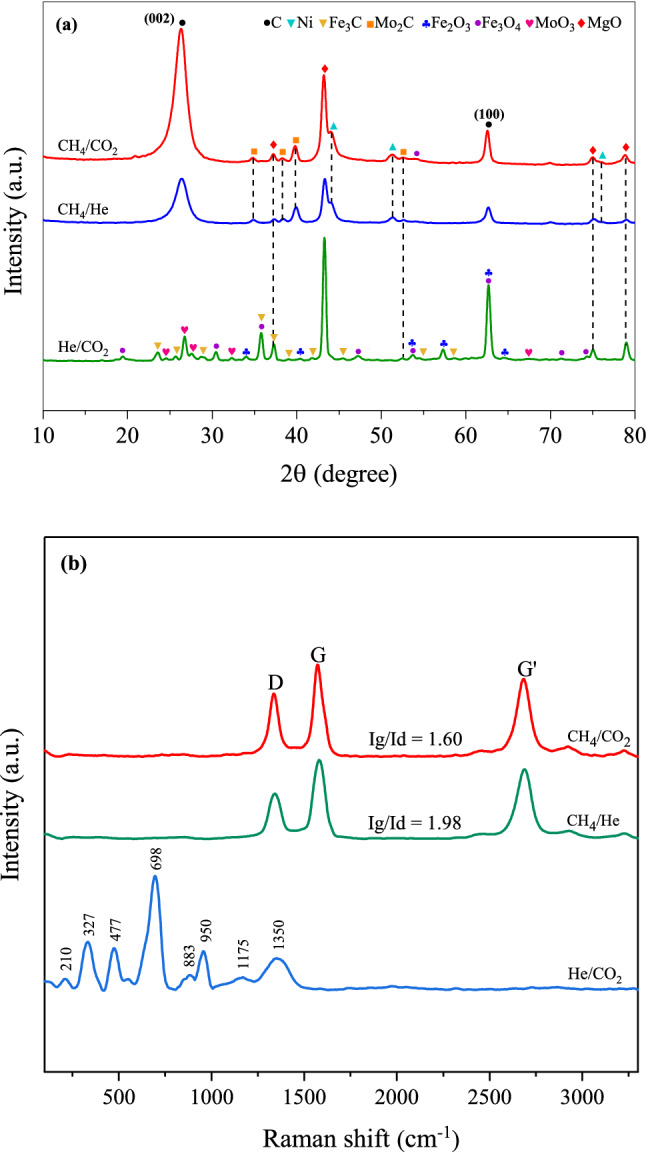
(**a**) XRD patterns and (**b**) Raman spectra of the samples after the reaction over FeNiMo/MgO catalyst at 800 °C for 3 h.

Figure 11a shows the XRD pattern over FeNiMo/MgO catalyst under CH_4_/CO_2_, CH_4_/He, and He/CO_2_ after the reaction. Some diffraction peaks attributed to Fe_2_O_3_, Fe_3_O_4_, and Fe_3_C were found in CH_4_/CO_2_ and He/CO_2_ conditions. In the case of He/CO_2_, the presence of Fe_2_O_3_ and Fe_3_O_4_ can be confirmed from the Raman spectra at 200–1400 cm^−1^, as seen in Fig. 11b. Meanwhile no characteristic peaks devoted to Fe_2_O_3_, Fe_3_O_4_, and Fe_3_C species were observed as to CH_4_/He condition. These findings assure that operating under the existence of Fe on the catalyst suffered from CO_2_ oxidation resulting in deactivation. The graphitization of CNTs produced by the mean of CH_4_ decomposition are shown in Fig. 11b. The results show that the synthesized CNTs on CH_4_/He had a higher graphitization degree of carbon structure (Ig/Id = 1.98) in comparison to that obtained by operating at normal CH_4_/CO_2_ condition (Ig/Id = 1.60). This may be explained by the decrease in the proportion of Fe metallic phase due to it can be oxidized into Fe oxides with CO_2_, corresponding to the lower level of graphitization in CNTs^47^.

**Figure 12:**
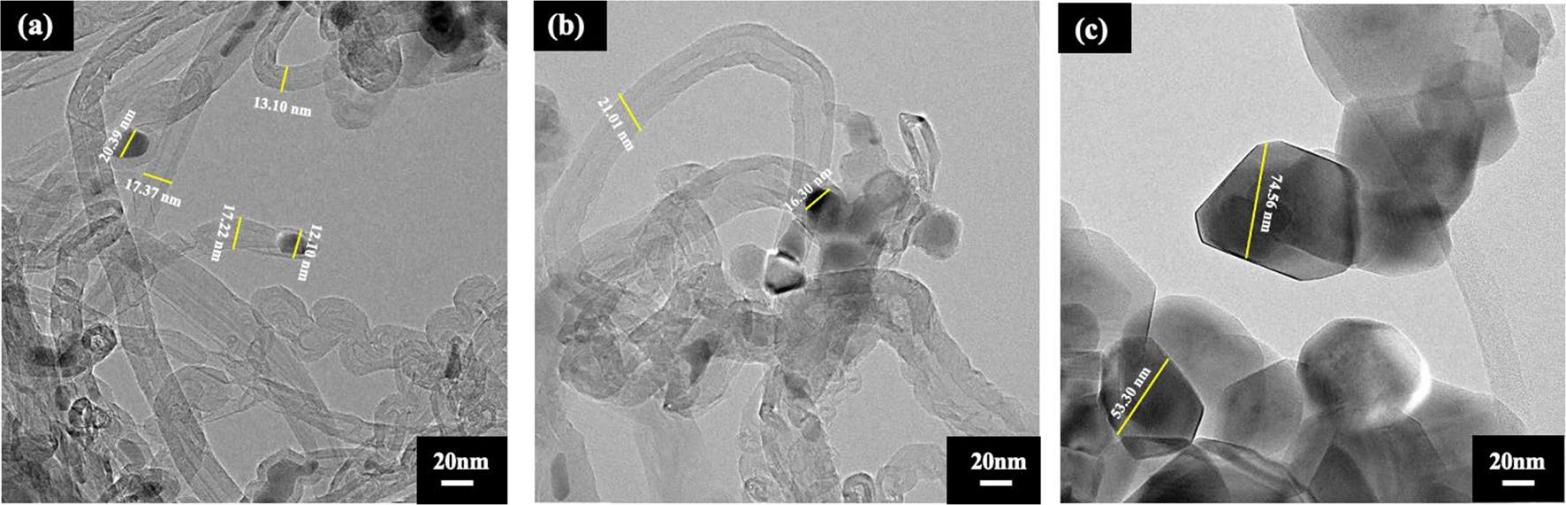
HR**-**TEM micrographs of the samples after running at 800 °C for 3 h over FeNiMo/MgO catalyst under (**a**) CH_4_/He, (**b**) CH_4_/CO_2_, (**c**) He/CO_2_, GHSV = 48,000 ml/gCat-h.

To further observe the catalyst oxidation by CO_2_ in biogas (CO_2_ + CH_4_), the two experiments of sole CO_2_ and sole CH_4_ balanced with He were carried out as shown in Fig. 12. Figure 12c reveals that the CO_2_ causes higher degree of oxidation, resulting in the transformation of metal into larger metal oxide particles. The formation of metal oxide particles regards as another key factor that is correlated with CNTs diameter. Thus, the synthesized CNTs using FeNiMo/MgO catalysts had enlarged sizes of CNTs. This is evident by TEM analysis as shown in Fig. 5.

## Conclusion

In this study, the bimetallic NiMo, trimetallic CoNiMo, FeNiMo, and CoFeMo supported on MgO were used as catalysts for CNTs growth and syngas production. The experimental results show that the introduction of the third composition (Co or Fe) to NiMo/MgO catalyst does not give any further increase in the catalytic performance. The NiMo/MgO catalyst can perform the highest activity for both reforming and methane cracking. In addition, only slight deactivation of NiMo/MgO catalyst was observed over 3 h. The high purity of syngas was obtained, while the narrowest distribution and the smallest diameter of CNTs with graphitization degree are comparable to those of commercial CNTs. This makes NiMo/MgO a remarkably outstanding catalyst to directly convert biogas into syngas and multi-walled carbon nanotubes. In the case of the Fe-containing catalysts, they are known to catalyze the carbon oxidation, causing the agglomeration of oxide phases, and the formation of large diameter CNTs. However, the existence of Fe in the catalyst greatly enhances the bamboo structure with high grades which may be useful in some practical applications.

## Data Availability

All data related to the finding of this study are accessible upon request from the corresponding author Sakhon Ratchahat.
